# Hydroxychloroquine Does Not Increase the Risk of Cardiac Arrhythmia in Common Rheumatic Diseases: A Nationwide Population-Based Cohort Study

**DOI:** 10.3389/fimmu.2021.631869

**Published:** 2021-04-02

**Authors:** Chien-Hsien Lo, James Cheng-Chung Wei, Yu-Hsun Wang, Chin-Feng Tsai, Kuei-Chuan Chan, Li-Ching Li, Tse-Hsien Lo, Chun-Hung Su

**Affiliations:** ^1^ Institute of Medicine, School of Medicine, Chung Shan Medical University, Taichung, Taiwan; ^2^ Division of Cardiology, Department of Internal Medicine, Chung-Shan Medical University Hospital, Taichung, Taiwan; ^3^ Division of Allergy, Immunology and Rheumatology, Chung Shan Medical University Hospital, Taichung, Taiwan; ^4^ Institute of Medicine, College of Medicine, Chung Shan Medical University, Taichung, Taiwan; ^5^ Graduate Institute of Integrated Medicine, China Medical University, Taichung, Taiwan; ^6^ Department of Medical Research, Taichung Veterans General Hospital, Taichung, Taiwan; ^7^ Department of Medical Research, Chung Shan Medical University Hospital, Taichung, Taiwan; ^8^ Department of Internal Medicine, Chung-Shan Medical University Hospital, Taichung, Taiwan; ^9^ Department of Internal Medicine, Da Chien General Hospital, Miaoli, Taiwan

**Keywords:** Hydroxychloroquine, arrhythmia, rheumatoid arthritis, systemic lupus erythematosus, Sjögren's syndrome

## Abstract

**Objectives:**

Hydroxychloroquine (HCQ) is widely used to treat rheumatic diseases including rheumatoid arthritis (RA), systemic lupus erythematosus (SLE) and Sjögren’s syndrome (SS). Cardiac arrhythmia has been concerned as important safety issue for HCQ. The aim of this study was to investigate whether hydroxychloroquine increases new-onset arrhythmia among patients with RA, SLE or SS.

**Methods:**

This was a retrospective cohort study that conducted from the longitudinal health insurance database of Taiwan. Patients with newly diagnosed RA, SLE or SS with age ≥20 years old were selected from 2000 to 2012. Patients who received HCQ and without HCQ treatment groups were matched by propensity score to minimize the effect of selection bias and confounders. The Cox proportional hazard model was used to analyze the risk of arrhythmia between the two groups after controlling for related variables.

**Results:**

A total of 15892 patients were selected to participate and finally 3575 patients were enrolled in each group after matching. There was no different risk of all arrhythmia in patients using HCQ than without HCQ (adjusted hazards ratio 0.81, 95% CI 0.61–1.07) and ventricular arrhythmia as well. The incidence of arrhythmia did not increase when HCQ co-administrated with macrolides. The arrhythmia risk was also not different regardless of daily HCQ dose <400mg or ≥400mg or follow-up duration of ≦4 months or >4 months.

**Conclusion:**

The administration of HCQ did not increase the risk of all cardiac arrhythmia and ventricular arrhythmia regardless of different duration of treatment (≦4 months or >4 months) or cumulative dose (<400mg or ≥400mg) in patients with common autoimmune diseases such as RA, SLE and SS.

## Introduction

Hydroxychloroquine (HCQ) is an antimalarial drug that also has been extensively used in certain rheumatic diseases such as rheumatoid arthritis (RA), systemic lupus erythematosus (SLE) and Sjögren’s syndrome (SS) to control disease activity and improve survival for several decades (1–5). HCQ can modulate prothrombotic signaling pathways and protect against systemic inflammation by inhibiting endosomal NADPH oxidase (6). Coronavirus disease 2019 (Covid-19) become pandemic since 2019 December, currently still spreads rapidly, rising number of cases and deaths worldwide (7, 8). Because of Covid-19 pandemic, the efficacy and safety of HCQ has been more concerned and explored recently. Cardiac arrhythmia is one of the safety issues in patients receiving HCQ. Previous studies had been debating on the effect of HCQ and the risk of cardiac arrhythmia (9–11). A recent meta-analysis including 45 articles concluded that HCQ use was not associated with mortality, benefit or harm in Covid-19 patient (12). A prospective trial reported that prolongation of QTc interval was found more frequently in the HCQ group, but there was no increase in arrhythmia (13). Rosenberg et al. reported more patients in the HCQ experienced arrhythmias compared with non-HCQ group (14). McGhie et al. pointed that cumulative antimalarial dose did not significant associate with ECG structural abnormalities, while was protective for ECG conduction abnormalities (15). Recent study for hospitalized patients showed that the risk of supraventricular tachycardia, ventricular arrhythmia and AV block were not increased in HCQ group (16). The data about the treatment of HCQ and clinical outcome are mixing and still inconsistent. Therefore, we designed a retrospective cohort study from large population-based dataset to investigate whether HCQ increase the risk of arrhythmia or not in patients with rheumatic diseases including RA, SLE and SS.

## Materials and Methods

### Data Source

We conducted a retrospective cohort study using data from the Taiwan National Health Insurance Research Database (NHIRD), which contains information on outpatient visits, emergency care, hospitalization, medical procedures, and medications. It contains one million people randomly sampled from the NHIRD with International Classification of Diseases, 9th Revision, Clinical Modification (ICD-9-CM) diagnosis codes (17) and data were collected from 1999 to 2013. This study was approved by the Research Ethics Committee of Chung Shan Medical University and Hospital (CS-17114).

### Patient Selection

This data was collected from 1999 to 2013 from the Longitudinal Health Insurance Database. We enrolled all people with age ≧20 years-old, who were newly diagnosed with RA (ICD-9-CM=714.0) (18, 19), SLE (ICD-9-CM=710.0) (6, 20) and SS (ICD-9-CM=710.2) with at least three outpatient clinic visits or one admission from 2000 to 2012. Patients with a diagnosis of arrhythmia (ICD-9-CM=426–427), before the diagnosis of disease RA, SLE or SS were excluded. The first date of HCQ after a disease diagnosis was set as the index date. The comparison group was non-use of HCQ after disease diagnosis. The comparison group included patients who had not been diagnosed with arrhythmia after the disease diagnosis. The primary outcome was defined as a new diagnosis of all arrhythmias including conduction disorders such as atrioventricular block and bundle branch block (ICD-9-CM=426), supraventricular tachyarrhythmias, ventricular tachyarrhythmias and sinus node dysfunction (ICD-9-CM=427). Ventricular tachyarrhythmias alone (ICD-9-CM=427.1, 427.4–427.5) was also analyzed as secondary outcome. The study period was from the index date to the first onset of arrhythmia, withdraw from the national health insurance system, or 2013/12/31, which came first.

### Covariates and Propensity Score Matching

To minimize the effect of confounding factors, we used propensity score (PS) matching to obtain a 1:1 matched by the age, gender, comorbidities such as hypertension (HTN: ICD-9-CM=401–405), hyperlipidemia (ICD-9-CM=272.0–272.4), chronic liver disease (ICD-9-CM=571), chronic kidney disease (CKD: ICD-9-CM=585), diabetes mellitus (DM: ICD-9-CM=250), chronic obstructive pulmonary disease (COPD: ICD-9-CM=491,492,496), ischemic heart disease (ICD-9-CM=410–414), heart failure (ICD-9-CM=428), stroke (ICD-9-CM=430–438), history of beta (β)-blocker usage, antibiotic macrolides treatment and index year. The pre-existing comorbidity was diagnosed one year before index date. The medications were used during the study period. PS matching is a statistical matching technique that can reduce potential confounding caused by unbalanced covariates in non-experimental settings. PS matching is the probability calculated *via* a logistic regression model. The score is a unit with certain characteristics, which can be used to reduce or eliminate selection bias.

### Statistical Analysis

To compare the characteristics of HCQ and non-HCQ groups, Chi-square test for categorical variables and independent t test for continuous variables were used. The incidence of events was determined by the number of events divided by the observed person-years. The Kaplan-Meier method was applied to obtain the cumulative incidences of newly diagnosed arrhythmia and log-rank test to perform the significance. We used a Cox proportional hazard model to estimate the crude hazard ratios (HR), adjusted HR, and 95% confidence intervals (CIs) among the two groups. The per-day HCQ dosage was also calculated to perform the risk of arrhythmia. All statistical analyses were conducted using software SPSS version 18.0 (SPSS Inc., Chicago, IL, USA). A p value less than 0.05 between the two groups was considered to be statistically significant.

## Results

**Figure 1:**
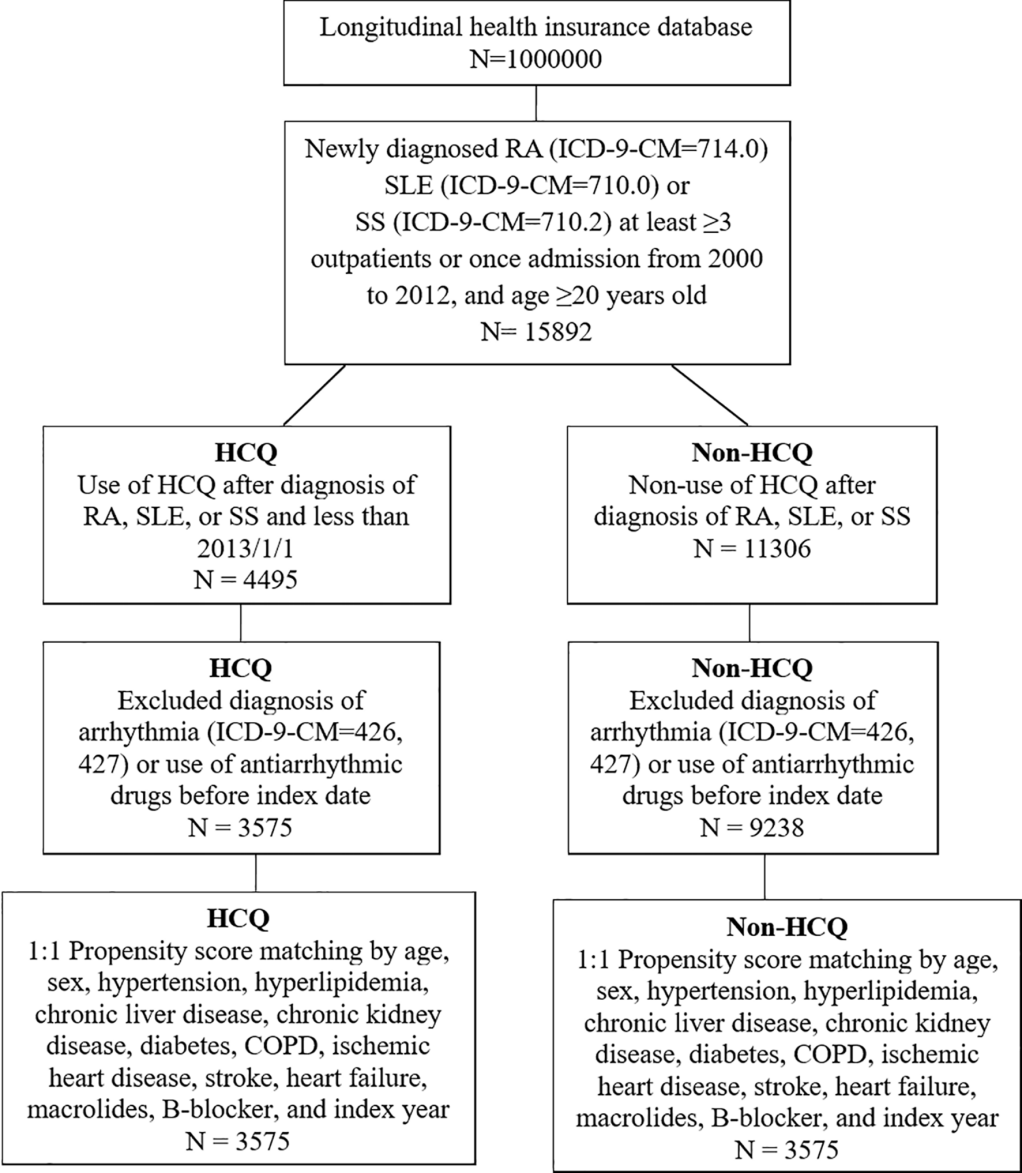
Flow chart of patient selection for those with rheumatoid arthritis (RA), systemic lupus erythematosus (SLE) or Sjögren’s syndrome (SS), who were using hydroxychloroquine (HCQ) (study group) and or not using HCQ (non-HCQ control group) from the National Health Insurance Research Database.

The flow chart for patient selection is demonstrated in **Figure 1**. Among the one million patients, a total of 15892 patients with newly diagnosed RA, SLE and SS were selected to participate in the study. 2988 patients were excluded due to previous arrhythmia before their RA diagnosis, those using antiarrhythmic agents such as amiodarone, dronedarone, or propafenone. After PS matching with 1:1 ratio, a total of 3575 patients were enrolled in the both groups respectively.

Baseline demographic and clinical characteristics of the participants are summarized in **Table 1**. The mean (SD) age of the patients was 51.1 (13) years in the both groups. About 80% of the study population was female. Most underlying comorbidities were higher in non-HCQ group which were balanced after PS matching. 438 (12.3%) used a macrolide antibiotic combined with HCQ, whereas 420 (11.7%) patients used macrolide antibiotics in the non-HCQ group (p=0.512).

**Table 1:** Demographic characteristics of the HCQ and Non-HCQ groups.

	Before PS matching					After PS matching				
	HCQ (N= 3575)		Non-HCQ (N = 9238)			HCQ (N= 3575)		Non-HCQ (N = 3575)		
	n	%	n	%	p-value	n	%	n	%	p-value
Age					<0.001					0.255
<50	1647	46.1	3786	41.0		1647	46.1	1695	47.4	
≥50	1928	53.9	5452	59.0		1928	53.9	1880	52.6	
Mean ± SD	51 ± 15.1		53.8 ± 15.9		<0.001	51 ± 15.1		51 ± 15.6		0.937
Sex					<0.001					0.720
Female	2873	80.4	6271	67.9		2873	80.4	2885	80.7	
Male	702	19.6	2967	32.1		702	19.6	690	19.3	
Hypertension	620	17.3	2094	22.7	<0.001	620	17.3	595	16.6	0.431
Hyperlipidemia	271	7.6	720	7.8	0.685	271	7.6	270	7.6	0.964
Chronic liver disease	206	5.8	481	5.2	0.211	206	5.8	203	5.7	0.879
Chronic kidney disease	49	1.4	105	1.1	0.276	49	1.4	49	1.4	1
Diabetes mellitus	274	7.7	941	10.2	<0.001	274	7.7	262	7.3	0.590
COPD	103	2.9	318	3.4	0.110	103	2.9	114	3.2	0.448
Ischemic heart disease	159	4.4	504	5.5	0.021	159	4.4	171	4.8	0.499
Stroke	96	2.7	345	3.7	0.003	96	2.7	99	2.8	0.828
Heart failure	33	0.9	99	1.1	0.455	33	0.9	40	1.1	0.410
Macrolides	438	12.3	1275	13.8	0.021	438	12.3	420	11.7	0.512
B-blocker	645	18.0	1789	19.4	0.087	645	18.0	612	17.1	0.305

HCQ, Hydroxychloroquine; PS, propensity score; COPD, Chronic obstructive pulmonary disease.

**Figure 2:**
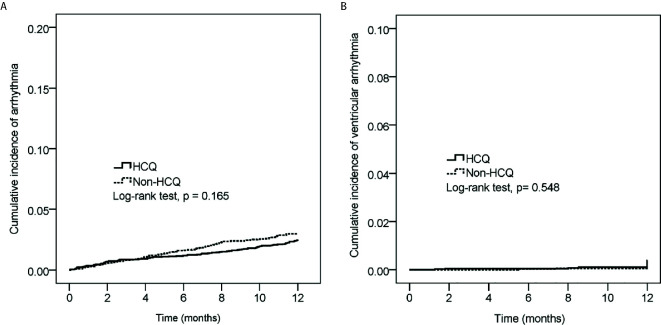
The cumulative incidence of all cardiac arrhythmia **(A)** and ventricular tachyarrhythmia **(B)** between the hydroxychloroquine (HCQ) group and the non-HCQ group (p-value=0.165 and p-value=0.548 respectively, Log-rank test).

The main arrhythmias outcome was illustrated in **Figure 2**. The cumulative risk of both all arrhythmia (**Figure 2A**) and ventricular tachyarrhythmia (**Figure 2B**) were not different in the HCQ group compared with non-HCQ group (log-rank test, p=0.165 and p=0.548 respectively).

The result of Cox regressions to determine the hazard ratios for arrhythmia is listed in **Table 2**. The incidence of arrhythmia did not increase in HCQ group with an adjusted HR of 0.81, 95% CI 0.61–1.07. The incidence of arrhythmia was 106 per 41916 person-months in the non-HCQ group and 87 per 42057 person-months in the HCQ group (**Table S1**). There was no different in ventricular arrhythmia in HCQ usage than in the non-HCQ group with an adjusted HR of 1.35, 95% CI 0.61–2.99 (**Table S2**). Age ≥50 years had higher risk of arrhythmia (adjusted HR 1.64, 95% CI 1.18–2.28). People with underlying hypertension, chronic kidney disease, ischemic heart disease, stroke and using β-blocker had significantly higher numbers of arrhythmias. Other comorbidities were not significantly different for the risk of arrhythmia.

**Table 2:** Association of arrhythmia in RA, SLE or SS patients with multivariable analysis and Cox proportional hazard analysis.

	Crude HR (95% C.I.)	p-value	Adjusted HR^†^ (95% C.I.)	p-value
HCQ				
No	Reference		Reference	
Yes	0.82 (0.62-1.09)	0.165	0.81 (0.61-1.07)	0.137
Age				
<50	Reference		Reference	
≥50	2.07 (1.52-2.82)	<0.001	1.64 (1.18-2.28)	0.003
Sex				
Female	Reference		Reference	
Male	1.01 (0.71-1.45)	0.940	0.95 (0.66-1.36)	0.760
Hypertension	1.58 (1.14-2.20)	0.006	0.61 (0.41-0.89)	0.011
Hyperlipidemia	2.01 (1.34-3.02)	<0.001	1.44 (0.93-2.22)	0.102
Chronic liver disease	1.20 (0.68-2.11)	0.522	0.92 (0.52-1.62)	0.765
Chronic kidney disease	4.22 (2.24-7.98)	<0.001	2.78 (1.42-5.42)	0.003
Diabetes	1.45 (0.91-2.30)	0.117	0.82 (0.50-1.34)	0.420
COPD	2.20 (1.23-3.95)	0.008	1.65 (0.90-3.02)	0.104
Ischemic heart disease	2.99 (1.95-4.58)	<0.001	1.65 (1.03-2.64)	0.036
Stroke	3.08 (1.82-5.22)	<0.001	2.00 (1.16-3.47)	0.013
Heart failure	3.46 (1.54-7.81)	0.003	1.50 (0.63-3.54)	0.361
Macrolides	0.94 (0.60-1.46)	0.778	0.80 (0.51-1.25)	0.329
B-blocker	3.87 (2.91-5.14)	<0.001	3.53 (2.59-4.80)	<0.001

HCQ, Hydroxychloroquine; RA, Rheumatoid arthritis; SLE, systemic lupus erythematosus; SS, Sjögren’s syndrome; HR, hazard rati; COPD, Chronic obstructive pulmonary disease;

^†^Adjusted for age, gender, hypertension, hyperlipidemia, chronic liver disease, chronic kidney disease, diabetes mellitus, COPD, ischemic heart disease, stroke, heart failure, macrolides, and B-blocker.

**Figure 3:**
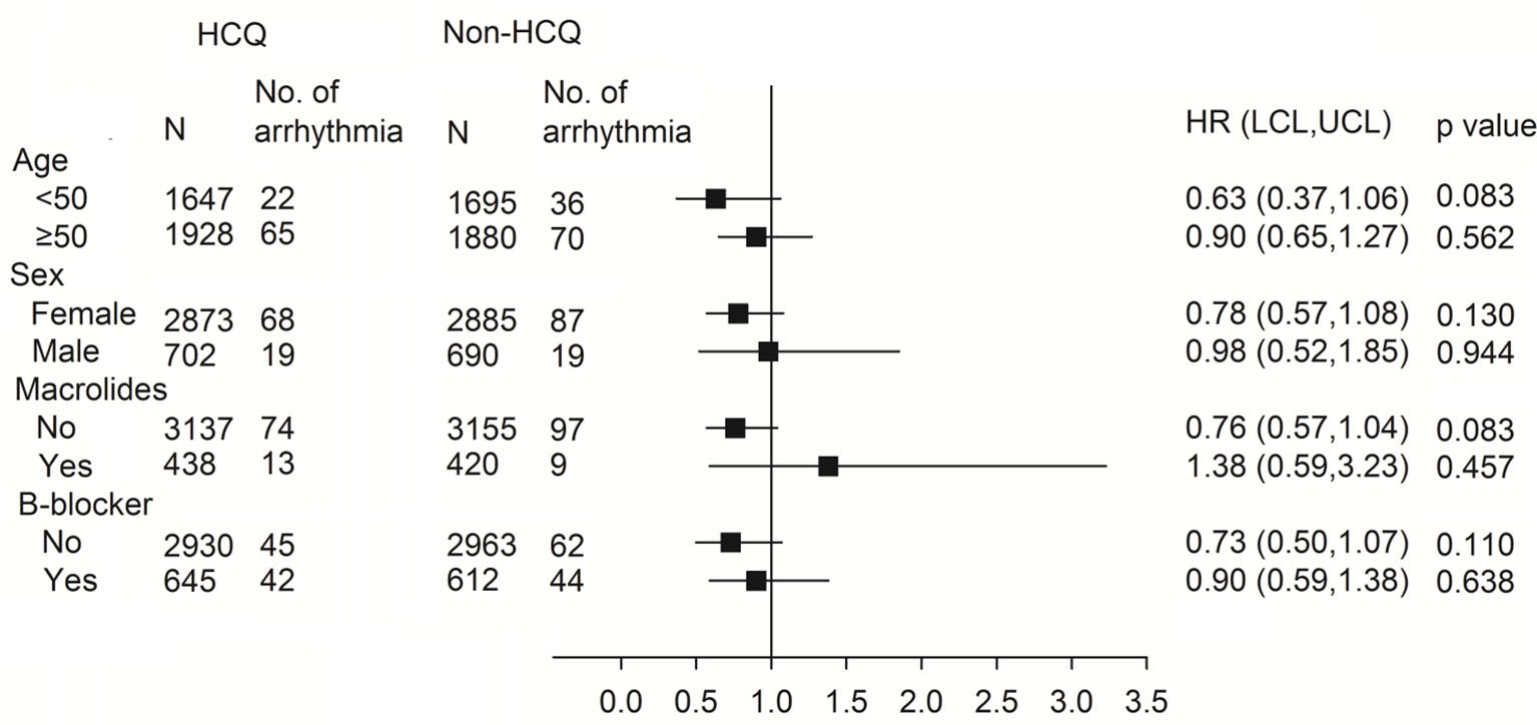
Subgroup analysis using the Cox proportional hazard model for the association between arrhythmia and HCQ. HR, hazard ratio; HCQ, hydroxychloroquine.

**Figure 4:**
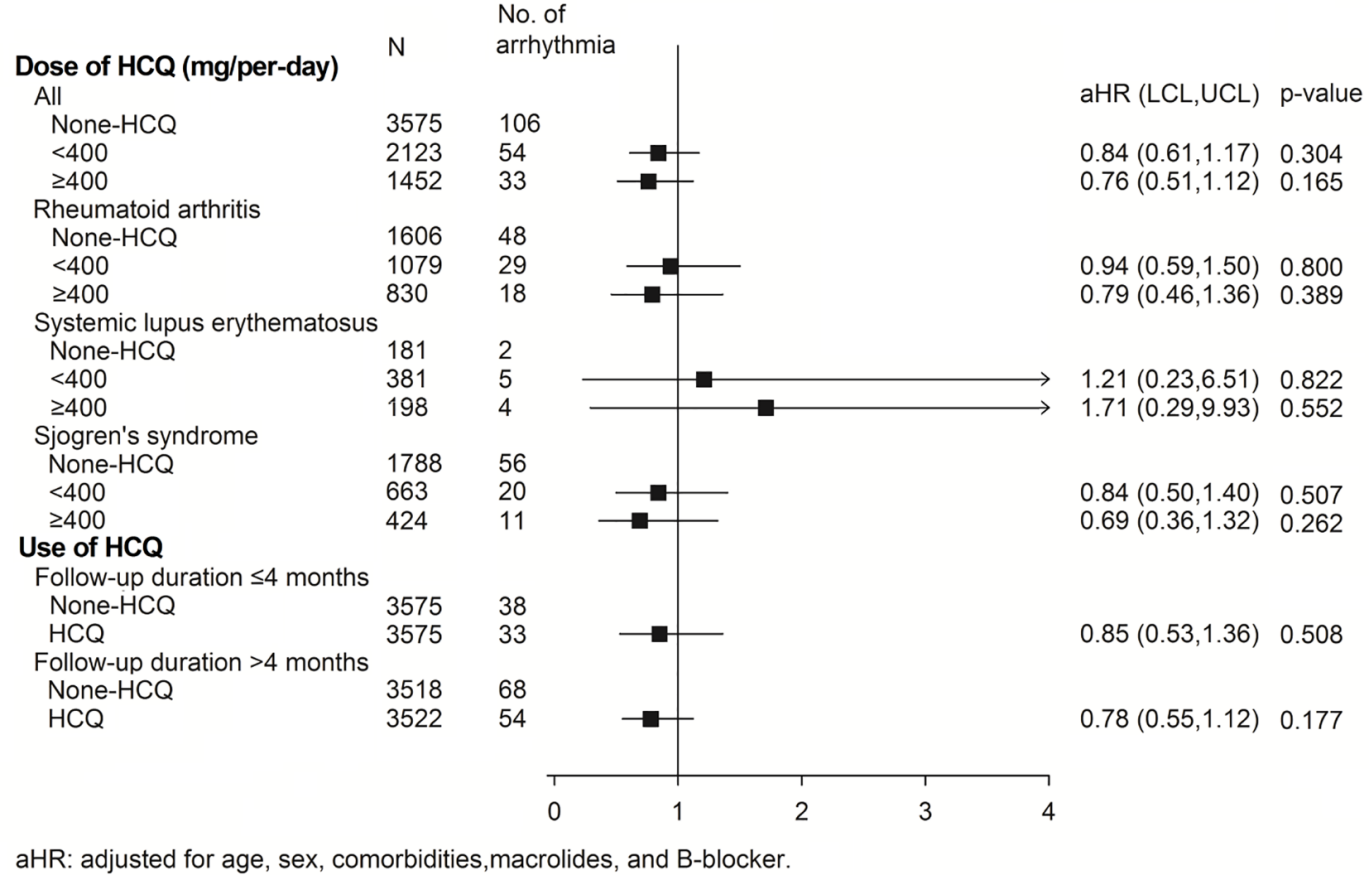
The risk of arrhythmias in the subgroup analysis of individual diseases and those with different daily HCQ doses and follow-up durations. aHR, adjusted hazard ratio; HCQ, hydroxychloroquine.

The subgroup analysis for association of arrhythmia between HCQ and non-HCQ group is shown in **Figure 3** and **Table S3**. There was no statistically significant difference between the combination of HCQ and macrolide antibiotics and incidence of arrhythmia (only 13 events over 438 patients in HCQ group compared with 9 events among 420 patients in the non HCQ group) (adjusted HR of 1.38, 95% CI 0.59–3.23). Age above or below 50 years, gender and β-blocker usage also did not increase the risk of arrhythmia either patients using HCQ or not. **Figure 4**, **Tables S4**, **S5** provide the Cox regression hazard ratios for the relationships between arrhythmia and HCQ cumulative dose. All three diseases as well as individual RA, SLE and SS were also analyzed. We found that the risk of arrhythmia for HCQ did not significantly different regardless of the daily dose of <400 mg (adjusted HR 0.84, 95% CI 0.61–1.17) or ≥400 mg (adjusted HR 0.76, 95% CI 0.51–1.12), and follow-up duration of ≦4 months (adjusted HR 0.85, 95% CI 0.53–1.36) or >4 months (adjusted HR 0.78, 95% CI 0.55–1.12) compared with non HCQ usage. The different dose result was also consistent in all sub-analyzed individual disease.

## Discussion

The aim of this study was to determine the safety concern of HCQ treatment and the risk of arrhythmia. The results indicated that common rheumatic disease patients using HCQ did not have higher risks of all kinds of cardiac arrhythmias including ventricular tachyarrhythmia. Furthermore, the risk of arrhythmia was not dependent on a longer duration of HCQ treatment >4 months or higher daily dose of ≥400mg.

HCQ has been used in many thousands of patients all over the world for the treatment of Covid-19 in early outbreak. However, recently observational and prospective studies have reported on the efficacy of HCQ treatment for Covid-19. Although some retrospective data disclosed use of HCQ alone and in combination with azithromycin decreased the Covid-19 associated mortality (21), most reports showed that HCQ treatment did not improve their clinical status or reduce the risk of intubation or death (13, 14, 16, 22). A recent systemic review and meta-analysis conducted by Putman et al. who collected 45 articles including 4 randomized controlled trials, 29 cohort studies and 12 case series, concluded that HCQ use was not associated with benefit or harm with regard to Covid-19 mortality (12). In addition, the safety of HCQ treatment in cardiac arrhythmia also had been discussed and results were inconsistent. Cavalcanti et al. conducted a prospective trial with 667 patients (13), and reported that prolongation of QTc interval was found more frequently in the HCQ group, but there was no increase in arrhythmia. Rosenberg et al. investigated 1438 hospitalized patients (14), reported 16% of patients in the HCQ experienced arrhythmias compared with 10% in the non-HCQ group. McGhie et al. analyzed 453 patients treated with antimalarial including HCQ and chloroquine which showed that cumulative antimalarial dose did not significant associate with ECG structural abnormalities, while was protective for ECG conduction abnormalities (15). Recent study from the RECOVERY collaborative group with 4716 hospitalized patients (16) showed that the risk of supraventricular tachycardia, ventricular arrhythmia and AV block were not increased in HCQ group. The study design, relatively small sample size and low event rate may have affected the outcomes and results. In the present study, we found no significant different between cardiac arrhythmia and the use of HCQ in most rheumatic patients.

An open-label trial discloses azithromycin added to HCQ was significantly more efficient for virus elimination (23). HCQ with or without azithromycin were used as a treatment option for Covid-19 in several countries during early outbreak (23, 24). However, co-administration of HCQ with other drugs such as azithromycin might amplify the arrhythmia risk. Some antibiotics, including macrolides, show pharmacodynamic evidence of iKr inhibition, which results in QT prolongation and dispersion of recovery across the ventricular wall. This phenomenon also has the chance to induce TdP and increase the risk of cardiovascular death (25, 26). In animal studies, there are no synergistic arrhythmic effects of azithromycin with or without chloroquine (27). Nevertheless, one retrospective cohort study reported that patients taking azithromycin had increased risk of mortality, including cardiovascular death, compared with those without antibiotic use during the 5 days of therapy (26). Several studies report the combination of HCQ and azithromycin in Covid-19 treatment prolonged the QT interval, however inconsistent outcomes about the development of life-threatening arrhythmia and mortality were found (13, 28–30). Hence, we also analyzed whether arrhythmia increased or not in the specific subgroups using HCQ with added macrolide treatment for other reasons, which showed that combination therapy of HCQ with macrolides also did not increase any arrhythmia (**Figure 3** and **Table S3**). Nonetheless, the event numbers (arrhythmias) are low in both the HCQ and non-HCQ groups. Even so, it may support the safety of HCQ combined with macrolide therapy.

Regarding comorbidities (**Table 2**), patients with CKD, ischemic heart disease and stroke had a significantly increased risk of arrhythmia. The anticipated result of CKD come from CKD related various arrhythmogenic alternation including autonomic nervous system, metabolic hemostasis, pharmacokinetics and pharmacodynamics of drugs that may facilitate cardiac arrhythmias (31, 32). β-blockers provide an antiarrhythmic effect because they decrease sympathetic activity by inhibition of the β1 adrenergic receptor and reduce atrial also ventricular tachyarrhythmia (33, 34). But they are also widely used in other cardiovascular diseases rather than arrhythmia such as post myocardial infarction, heart failure, and hypertension (35, 36). In our study, β-blockers were matched by PS matching to reduce possible confounding bias. We found that patients taking β-blocker developed more arrhythmia with an adjusted hazard ratio of 3.53. It may be due to patients taking β-blocker had more underlying comorbidities. In the subgroup analysis, β-blocker did not further increase or decrease risk of arrhythmia either patients using HCQ or not.

Recently we have reported that patients with RA using HCQ did not have a higher risk of cardiac arrhythmia regardless of the daily HCQ dose or follow-up duration (37), which result is consistent with the current study. In addition to RA population, our study includes SLE and SS which increase the sample size, better matching between the 2 groups and further subgroup analysis. We also determined the sensitivity analysis for development of only ventricular tachyarrhythmia as secondary outcome, which also disclosed consistent outcome. The sub-group analysis of the individual disease of RA, SLE and SS, all revealed no significant increase risk of arrhythmia. Our data showed that the administration of HCQ had a neutral effect on the development of all arrhythmias and ventricular tachyarrhythmias. The previous study reports cardiotoxicity of HCQ was possibly associated with cumulative dose (38). The chronic use of HCQ has also been reported to provoke cardiac arrhythmia (10). According to the sub-analysis (**Figure 4**, **Tables S4** and **S5**), we did not find the incidence of cardiac arrhythmia increase with larger HCQ dose or longer follow-up duration. These data suggest the safety of HCQ regardless of different dose (<400mg or ≥400mg) or treatment duration (≦4 months or >4 months).

There are some strengths and limitations in our study. The strength of this study stands its large NHIRD system, covers 99% of the Taiwanese population, which minimize bias from selection, poor recall or participation (39). Limitations were also notified. First, electrocardiography (ECG) is recommended to measure the QTc interval in individuals receiving HCQ administration (40). In the database, we could not determine the QTc interval by ECG. Nevertheless, our result is safe for risk of arrhythmia in HCQ therapy even we did not provide a baseline ECG indicated that routine ECG data is not always required. Second, only macrolide antibiotics were analyzed as combination therapy, and we did not further investigate other possible drugs that could have prolonged QT. Third, duration and dose of macrolides are not reported as the event numbers is less. Thus claims of association between HCQ and macrolides should be limited. Fourth, diseases were defined according to ICD-9 codified data without any procedure codes, which may lead to over diagnosis. Thence, we added disease codes with at least three outpatient clinic visits or one admission to reduce this bias. Fifth, our study population limited for the patients with RA, SLE and SS hence the results should not be totally applied on patients using HCQ for other diseases. Finally, this is a retrospective cohort design so patient follow-up is not by protocol. There may remain as the possibility of having mild cases of arrhythmias in either group which may have gone undetected. Further larger prospective or randomized control trials are necessary to verify the outcomes of this study.

## Conclusion

The administration of HCQ in patients with RA, SLE and SS did not increase the risk of cardiac arrhythmia as well as ventricular tachyarrhythmia regardless of the treatment duration (≦4 months or >4 months) or cumulative dose (<400mg or ≥400mg). The outcome is also consistent with others for combination HCQ with macrolide antibiotics.

## Data Availability Statement

The original contributions presented in the study are included in the article/**Supplementary Material**. Further inquiries can be directed to the corresponding author.

## Ethics Statement

The studies involving human participants were reviewed and approved by Research Ethics Committee of Chung Shan Medical University and Hospital (approval no. CS-17114). Written informed consent for participation was not required for this study in accordance with the national legislation and the institutional requirements.

## Author Contributions

C-HL and JC-CW designed the study, generated the figures and wrote the manuscript. Y-HW analyzed the data and generated the figures. C-FT, K-CC, L-CL and T-HL performed the bioinformatics analysis and wrote the manuscript. C-HS made substantial contributions to the design of the study, conducted the data analysis and figure generation, and wrote the manuscript. All authors contributed to the article and approved the submitted version.

## Conflict of Interest

The authors declare that the research was conducted in the absence of any commercial or financial relationships that could be construed as a potential conflict of interest.
