# Supplementary material for: Hydroxychloroquine Does Not Increase the Risk of Cardiac Arrhythmia in Common Rheumatic Diseases: A Nationwide Population-Based Cohort Study
Source: Front Immunol. 2021 Apr 2;12:631869. doi: 10.3389/fimmu.2021.631869 (PMC8050346; doi:10.3389/fimmu.2021.631869)
Supplement: Supplementary file 1 [file DataSheet_1.docx]

Table S1. Poisson regression of relative risk of HCQ and non-HCQ

|  | Non-HCQ | HCQ |
| --- | --- | --- |
| N | 3575 | 3575 |
| Person-months | 41916 | 42057 |
| No. of arrhythmia | 106 | 87 |
| ID (95% C.I.) | 2.53 (2.09-3.06) | 2.07 (1.68-2.55) |
| Relative risk (95% C.I.) | Reference | 0.82 (0.62-1.09) |

ID: incidence density (per 1000 person-months)

Table S2. Sensitivity analysis for the risk of ventricular tachyarrhythmia in hydroxychloroquine

(HCQ) and non-HCQ

|  | N | No. of ventricular tachyarrhythmia | Crude HR  (95% C.I.) | p-value | Adjusted HR^†^  (95% C.I.) | p-value |
| --- | --- | --- | --- | --- | --- | --- |
| Outcome: ventricular arrhythmia (ICD-9-CM=427.1,427.4,427.5) | | | |  |  |  |
| Group |  |  |  |  |  |  |
| Non-HCQ | 3575 | 11 | 1 |  | 1 |  |
| HCQ | 3575 | 14 | 1.27 (0.58-2.80) | 0.550 | 1.35 (0.61-2.99) | 0.460 |

†Adjusted for age, gender, comorbitidies and medications in Table 1

Table S3. Subgroup analysis with Cox proportional hazard model for association of arrhythmia and hydroxychloroquine (HCQ)

|  | HCQ | |  | Non-HCQ | |  |  |
| --- | --- | --- | --- | --- | --- | --- | --- |
|  | N | No. of arrhythmia |  | N | No. of arrhythmia | HR (95% C.I.) | p-value |
| Age |  |  |  |  |  |  |  |
| <50 | 1647 | 22 |  | 1695 | 36 | 0.63 (0.37-1.06) | 0.083 |
| ≥50 | 1928 | 65 |  | 1880 | 70 | 0.90 (0.65-1.27) | 0.562 |
| p for interaction= 0.248 | | | | | | | |
| Sex |  |  |  |  |  |  |  |
| Female | 2873 | 68 |  | 2885 | 87 | 0.78 (0.57-1.08) | 0.130 |
| Male | 702 | 19 |  | 690 | 19 | 0.98 (0.52-1.85) | 0.944 |
| p for interaction= 0.540 | | | | | | | |
| Macrolides | |  |  |  |  |  |  |
| No | 3137 | 74 |  | 3155 | 97 | 0.76 (0.57-1.04) | 0.083 |
| Yes | 438 | 13 |  | 420 | 9 | 1.38 (0.59-3.23) | 0.457 |
| p for interaction= 0.200 | | | | | | | |
| B-blocker |  |  |  |  |  |  |  |
| No | 2930 | 45 |  | 2963 | 62 | 0.73 (0.50-1.07) | 0.110 |
| Yes | 645 | 42 |  | 612 | 44 | 0.90 (0.59-1.38) | 0.638 |
| p for interaction= 0.467 | | | | | | | |

Table S4. Individual sub-analysis disease of the risk of arrhythmias on different daily hydroxychloroquine (HCQ) dose

|  | N | No. of arrhythmia | Crude HR (95% C.I.) | p-value |  | Adjusted HR^†^ | p-value |
| --- | --- | --- | --- | --- | --- | --- | --- |
| Dose of HCQ (mg/per-day) | | |  |  |  |  |  |
| None | 3575 | 106 | Reference |  |  | Reference |  |
| <400 | 2123 | 54 | 0.86 (0.62-1.19) | 0.357 |  | 0.84 (0.61-1.17) | 0.304 |
| ≥400 | 1452 | 33 | 0.76 (0.52-1.13) | 0.171 |  | 0.76 (0.51-1.12) | 0.165 |
| **Rheumatoid arthritis** | |  |  |  |  |  |  |
| Dose of HCQ (mg/per-day) | | |  |  |  |  |  |
| None | 1606 | 48 | Reference |  |  | Reference |  |
| <400 | 1079 | 29 | 0.90 (0.57-1.42) | 0.646 |  | 0.94 (0.59-1.50) | 0.800 |
| ≥400 | 830 | 18 | 0.72 (0.42-1.23) | 0.228 |  | 0.79 (0.46-1.36) | 0.389 |
| **Systemic Lupus Erythematosus** | | |  |  |  |  |  |
| Dose of HCQ (mg/per-day) | | |  |  |  |  |  |
| None | 181 | 2 | Reference |  |  | Reference |  |
| <400 | 381 | 5 | 1.17 (0.23-6.05) | 0.848 |  | 1.21 (0.23-6.51) | 0.822 |
| ≥400 | 198 | 4 | 1.79 (0.33-9.75) | 0.503 |  | 1.71 (0.29-9.93) | 0.552 |
| **Sjogren's syndrome** | |  |  |  |  |  |  |
| Dose of HCQ (mg/per-day) | | |  |  |  |  |  |
| None | 1788 | 56 | Reference |  |  | Reference |  |
| <400 | 663 | 20 | 0.96 (0.58-1.60) | 0.884 |  | 0.84 (0.50-1.40) | 0.507 |
| ≥400 | 424 | 11 | 0.83 (0.44-1.59) | 0.578 |  | 0.69 (0.36-1.32) | 0.262 |

†Adjusted for age, gender, comorbitidies and medications in Table 1

Table S5. Sensitivity analysis of follow-up duration

|  | N | No. of arrhythmia | Crude HR  (95% C.I.) | p-value | Adjusted  HR^†^ (95% C.I.) | p-value |
| --- | --- | --- | --- | --- | --- | --- |
| Follow-up duration ≤4 months | | | | | | |
| Group |  |  |  |  |  |  |
| Non-HCQ | 3575 | 38 | 1 |  | 1 |  |
| HCQ | 3575 | 33 | 0.87 (0.55-1.39) | 0.555 | 0.85 (0.53-1.36) | 0.508 |
| Follow-up duration >4 months | | | | | | |
| Group |  |  |  |  |  |  |
| Non-HCQ | 3518 | 68 | 1 |  | 1 |  |
| HCQ | 3522 | 54 | 0.79 (0.55-1.13) | 0.195 | 0.78 (0.55-1.12) | 0.177 |

HCQ: hydroxychloroquine

†Adjusted for age, gender, comorbitidies and medications in Table 1
